# Do cows see the forest or the trees? A preliminary investigation of attentional scope as a potential indicator of emotional state in dairy cows housed with their calves

**DOI:** 10.3389/fvets.2023.1257055

**Published:** 2023-09-28

**Authors:** Heather W. Neave, Jean-Loup Rault, Melissa Bateson, Emma Hvidtfeldt Jensen, Margit Bak Jensen

**Affiliations:** ^1^Department of Animal and Veterinary Science, Aarhus University, Tjele, Denmark; ^2^Institute of Animal Welfare Science, University of Veterinary Medicine Vienna, Vienna, Austria; ^3^Biosciences Institute, Newcastle University, Newcastle upon Tyne, United Kingdom

**Keywords:** affective state, cognitive bias, attention bias, mood, cow-calf contact

## Abstract

A positive mood in humans tends to broaden attentional scope while negative mood narrows it. A similar effect may be present in non-human animals; therefore, attentional scope may be a novel method to assess emotional states in livestock. In this proof-of-concept exploratory study, we examined the attentional scope of dairy cows housed with their calves either full-time, part-time (during daytime only), or with no calf contact (enrolled *n* = 10 each). Housing conditions were previously verified to induce differences in positive and negative emotional state, where part-time was considered more negative. Cows were trained to approach or avoid hierarchical images on a screen that were consistent in local and global elements (i.e., 13 small circles or crosses arranged in an overall circle or cross). After discrimination learning (>80% correct, over two consecutive days), 14 cows proceeded to test (*n* = 6 each full-and part-time; *n* = 2 no-contact, not analyzed). Test images showed inconsistent combinations of global and local elements (i.e., the overall global shape differs from the smaller local elements, such as a global circle composed of smaller local crosses and vice versa). Over two test days, approach responses to global and local images (each presented four times) were recorded. All cows were more likely to approach the local than the global image, especially part-time cows who never approached the global image; this may reflect a narrowed attentional scope in these cows. Full-time cows approached images more often than part-time cows, but overall response rates to global and local images were low, making specific conclusions regarding attentional scope difficult. Different housing conditions have potential to affect attentional scope, and possibly emotional state, of dairy cows, but statistical comparison to no-contact treatment was not possible. Cortisol concentration did not affect responses to images; thus arousal due to treatment or test conditions could not explain test performance. Further work with refined methodology and a larger sample size is required to validate the reliability of attentional scope as an assessment method of emotional state in cattle. Beyond this, the attentional scope test revealed how cattle may process, learn and respond to different visual hierarchical images, which further our understanding of cognitive and visual processes in cattle.

## 1. Introduction

The past decade has seen a rapid growth in work aimed at the objective assessment of emotional states of farm animals to evaluate animal welfare. The most common and validated approaches arise from our understanding of how emotions influence a range of cognitive processes, such as judgment, attention and memory (1, 2). This phenomenon is broadly referred to as cognitive bias, where the focus of most emotional state research in animals has been on judgment bias [referring to the propensity of an animal to respond more positively or more negatively when presented with ambiguous or uncertain information (3)]. Judgment bias tests appear to be sensitive to emotional valence (i.e., positive versus negative) rather than to arousal (i.e., high versus low intensity), making this methodology advantageous (2).

Attention biases are another type of cognitive bias in animals that have received much less research attention [but see review by Crump et al. (4)]. This type of bias refers to the differential allocation of attention to particular stimuli due to an animal’s emotional state. For instance, anxiety-induced sheep and beef cattle showed increased attention and vigilance behavior toward a threat (a dog) compared to control animals (5, 6), but more recent work indicates mixed results (7, 8). However, the time spent attending to a stimulus as a measure of attention bias may be difficult to interpret. Pigs managed in barren housing showed less attention toward a threat (flashing light, moving door, and loud noise) compared to those in enriched housing (9), and dairy heifers housed in negative experimentally-induced conditions attended to a threat (dog model) later than heifers in the positive or commercial standard housing conditions (10). These responses possibly reflect threat-avoidance rather than increased attention to the threat in animals in more negative states (11). Furthermore, dairy cows that were fed an energy restricted diet (leading to a state of hunger) did not show more attention toward a food source in comparison to cows fed a normal diet (12). These few studies in livestock suggest that attention bias could be a potentially useful indicator of emotional state, but the reliance on specific behaviors directed at a stimulus may be difficult to measure or interpret if results are not aligned with predictions.

A different approach to measuring attention bias is to examine how individuals respond to visual patterns, such as an image of a large shape formed from several smaller shapes. The test measures attentional scope by assessing whether individuals direct more attention to the broader visual field (the larger, global shape) or to the narrowed visual field (the smaller, local shapes) by using geometrical hierarchical visual stimuli (13). Early work by Navon (14, 15) demonstrated that humans predominantly attend to the global features of a visual scene before the local details of the scene (i.e., they see the “forest” over the “trees”). These perceptual processes in humans can be influenced by emotional state. For example, Gasper and Clore (16) showed subjects a target image composed of global and local elements (small triangles arranged in a larger triangle), and then asked subjects to indicate which of two images was most like the target image – one that matched the global shape (a triangle made up of smaller squares), or one that matched the local shape (a square made up of smaller triangles). Subjects that were in a positive-induced mood more often selected the global shape, while those in a negative-induced mood more often selected the local shape. This ‘broadening’ of attentional scope in positive individuals is thought to relate to the broaden-and-build theory whereby positive affect generates a broad cognitive style that is open, flexible, integrative and creative (17). In contrast, a “narrowing” of attentional scope in negative individuals may relate to devoting attention to specific threats and thus focusing on specific aspects of the visual field to eliminate irrelevant cues (18). However, more recent evidence suggests that attentional scope in humans may be driven by emotional intensity (i.e., arousal) rather than whether the emotional state is positive or negative (i.e., valence); low arousal states (such as feeling sad, calm, or relaxed) broadened attentional scope while high arousal states (such as feelings of fear, stress, or desire) narrowed attentional scope (19, 20). Overall, attentional scope in humans may be affected by both valence and arousal of emotional state [see review by Lacey et al. (21)], which deserves exploration in non-human animals.

The attentional scope test has been adapted for use in non-human species to evaluate differences in visual processing across animal taxa, including monkeys (22), dogs (23), splitfin fish (24), domestic chicks (25), pigeons (26) and honeybees (27). The methodology involves training animals to select a rewarded image and to avoid selecting an unrewarded or punished image. These images display shapes that are consistent in global and local elements, e.g., such as several small squares arranged to form a square. Then, animals are tested for their attention bias to global or local elements of the image by having the animal make a choice between images that are inconsistent in shape – the familiar small squares arranged in a new shape (local choice), or small new shapes arranged in the familiar square (global choice). Research to date tends to report a bias toward local processing in animals, although honeybees and fish showed a global bias (24, 27). The reason for differential bias among species can arise from ecological adaptations for visual processing of objects in the natural environment (24). In cattle, visual acuity is optimized horizontally in the retina, presumably for detecting predators at a distance on the horizon (28). However, large interspecies individual variation in bias was reported in dogs (23), suggesting that individual factors, such as emotional state of the animal, may contribute to a global or local attention bias. Only one recent study has examined if attentional scope is affected by emotional state in a non-human species (29). Using either food or social rewards, the authors induced four emotional states differing in arousal and valence in dogs; irrespective of arousal, dogs showed a narrowing of attentional scope in the positive food-reward-based treatments, but the opposite trend (narrowed attentional scope in the negative treatment) was observed in the social-reward-based treatment. Despite the mixed results, this study provides support that emotional state may influence attentional scope in animals.

In this proof-of-concept study, we aimed to assess whether dairy cows experiencing putatively different positive and negative emotional states show differences in attentional scope in a novel methodology for cattle. We housed dairy cows with their calves for two different daily durations: either full-time (23 h/d, except for milking times) or part-time (10 h/d during daytime, and cows were separated from their calves during nighttime). In our companion study using the same cows as reported in the present study, these housing conditions were verified to induce differences in emotional valence (30); part-time cows showed a negative (pessimistic) judgment bias, suggestive a negative emotional state relative to full-time cows but not to no-contact cows. Thus, we hypothesized that part-time and no-contact cows would show a local bias (narrowed attentional scope) in the attentional scope test, suggesting a negative emotional state compared to full-time cows. We expected this to be unrelated to arousal, assessed by measuring cortisol before and after the test.

## 2. Materials and methods

This study was conducted from September 2021 to February 2022 at the Danish Cattle Research Center, Aarhus University (Tjele, Denmark). All animal procedures were approved by the Danish Animal Experiments Inspectorate (Permit No. 2021-15-0201-00989) in accordance with the Danish Ministry of Environment and Food Act No. 474 (May 15, 2014).

### 2.1. Animal management and treatment groups

Thirty-six Danish Holstein dairy cows were enrolled at calving in 3 blocks of 12 cows. Cows calved in an individual maternity pen where they remained with their calf for approximately 48 h (range 42–66 h). Eligibility for enrolment in the study and assignment to treatment required the cow and calf to be healthy, calving without complication, no twin births, and that the calf was able to suckle from the cow without assistance within 48 h. Within block, cows and their calves were assigned to one of three housing treatments: (1) full-time contact between the cow and calf, apart from milking times (total 23 h/d cow-calf contact), (2) part-time contact between the cow and calf, between morning and afternoon milking at 0530 and 1530 h, and separation from the calf during the nighttime hours (10 h/d cow-calf contact), and (3) no-contact, where the cow was separated from the calf after leaving the maternity pen, and had no further contact with the calf. Assignment to treatments occurred in pairs (i.e., two cow-calf pairs, to minimize stress of entering a pen alone) until all 12 cows were assigned to a treatment group. Order of treatment assignment was pre-determined for each block, and rotated each block: part-time, full-time, no-contact (Block 1); full-time, no-contact, part-time (Block 2); no-contact, part-time, full-time (Block 3). Treatment within block thus contained four cows and their four calves, and were balanced for two primiparous and two multiparous cows whenever possible.

Full-time and part-time cows and their calves (*n* = 24) were housed in a dedicated barn containing 7.5 m × 9 m pens with straw bedding (4 cows and 4 calves of the same treatment, per pen). Calves were able to freely move around the pen among the cows, and also had exclusive access to two calf creep areas (3 × 3 m and 1.5 × 1.5 m) on the back left and right sides of the main pen containing *ad libitum* concentrate from a bowl and hay from a rack. The larger calf creep area also offered water from a self-filling bowl. Cows had access to two rotating grooming brushes mounted to the sides of the pen, and to two self-filling water bowls. Two feed troughs (each 2 × 0.75 m) provided cows *ad libitum* access to a TMR (approximately 50:50 concentrate to roughage ratio) replenished twice daily at 0800 and 2000 h; calves were also able to access this TMR. Straw bedding was added daily and completely cleaned out every 4 wk.

In a separate barn from the full-time and part-time cows, no-contact cows (*n* = 12) were housed in a pen of 8 experimental cows (4 per block) and 4 non-experimental cows, with no visual or auditory contact with their calves. Cows were fed a total mixed ration (TMR) twice daily at 1030 and 2000 h into 12 computerized feed bins (Insentec B.V., Marknesse, Netherlands). The pen was equipped with an automated rotating brush and 12 lying stalls with mattresses, which were topped with sawdust daily.

All cows were milked twice daily in a double 12 parallel milking parlor; full-time and part-time cows at 0500 and 1530 h, and no-contact cows 30 min later. Full-time cows always returned to their home pen after each milking, while part-time cows were re-directed after afternoon milking to a dedicated pen in a separate barn, without visual or auditory contact with their calves. This pen contained 14 lying stalls each equipped with a mattress and topped with sawdust daily, and fresh *ad libitum* TMR was delivered at 2000 h at a feed bunk with headlocks. After morning milking, part-time cows returned to their home pen with their calves.

### 2.2. Sample size and overview of the attentional scope test

Table 1 provides the number of cows in each treatment that completed each phase of the study. Due to management of workload for research staff, only 30 of the 36 enrolled cows were selected for training (*n* = 10 per treatment), selected based on entry order to the treatment pen (Block 1, *n* = 11; Block 2, *n* = 8; Block 3, *n* = 11). These cows were enrolled for training in the current study the day after entering the treatment pen, and had to complete the study within 25 days due to the start of a forthcoming experiment. There was also a concurrent experiment that tested cows in a judgment bias test before the attentional scope test [reported in Neave et al. (30)], which introduced an additional time constraint for completing the attentional scope test. Therefore, the number of cows that were able to complete the attentional scope test in due time was low, especially for no-contact cows (*n* = 2). Cows that did not complete in time were de-enrolled (see below for details).

**Table 1:** Number of dairy cows that were enrolled and completed each phase of training and testing for each treatment (full-time, part-time or no calf contact).^a^^,^^b^

Phase	Treatment			
	Full-time	Part-time	No-contact	Total
Enrolled	10	10	10	30
Completed initial training	9	8	10	27
Completed discrimination training	8	6	6	20
Tested in concurrent experiment (judgment bias test) before attentional scope^a^	8	6	6	20
Tested in attentional scope^a^	6	6	2	14
Tested in local testing^b^	5	2	1	6

Two concurrent experiments introduced time constraints for completing attentional scope testing in the required time frame.

a All cows that completed discrimination training were used in a concurrent experiment (judgment bias test) for 4 days, before the attentional scope test. At least 2 days were required to complete attentional scope testing before the start of the forthcoming experiment. Thus the difference in sample size between cows completing discrimination training and completing attentional scope is due to time constraints and not due to failure to learn the discrimination task.

b Sample size for local testing was further reduced due to time constraints. Only cows with available time remaining before the start of the forthcoming experiment went on to participate in the local testing.

**Figure 1:**
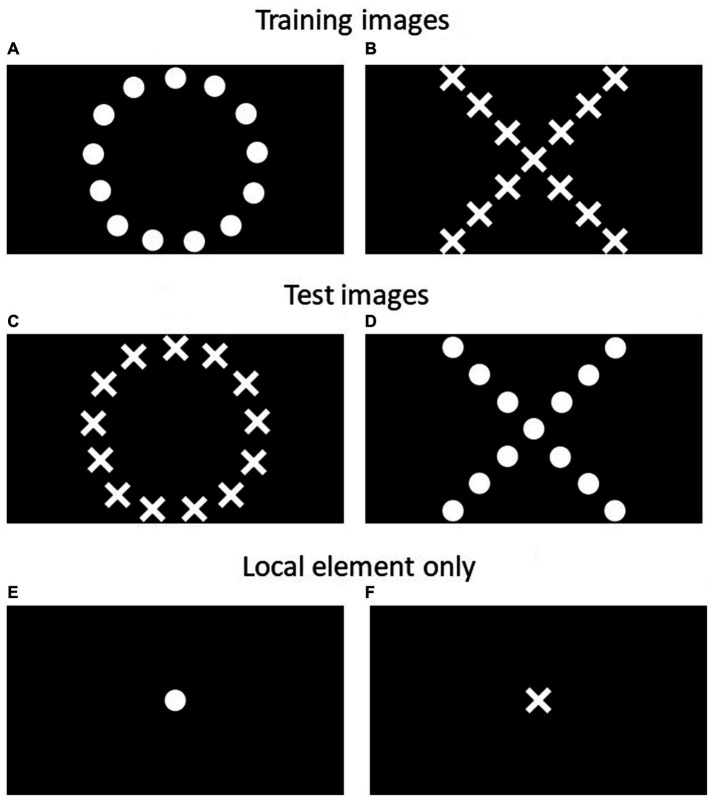
Images used in training and testing periods of the attentional scope task. Training images **(A,B)** are consistent in global and local elements; cows received either **(A)** or **(B)** as their positive image, and the opposite image was assigned as their negative image; Test images **(C,D)** are inconsistent in global and local elements. For cows trained with **(A)** as their positive image, **(C)** is the global image and **(D)** is the local image. For cows trained with **(B)** as their positive image, **(D)** is the global image and **(C)** is the local image; Local elements only **(E,F)** show only the local element of the positive and negative training images; cows trained with **(A)** as their positive image should approach **(E)**, and cows trained with **(B)** as their positive image should approach **(F)**.

An overview of the training and testing steps are in Table 2. Cows were first trained in a visual go/no-go discrimination task, followed by an attentional scope test, using methods adapted from Truppa et al. (24) for fish and Pitteri et al. (23) for dogs, who both used go/go tasks. A go/no-go task was used because these cows also participated in a judgment bias test which required a go/no-go training method. Briefly, cows were trained to approach a positive image to receive a food reward, and to avoid approaching a negative image to avoid receiving a punishment. The images consisted of 13 small white shapes (referred to as ‘local’ elements) spatially arranged to form a larger shape (referred to as “global” element), presented on a black background. These shapes were either small circles arranged in an overall circle, or small crosses arranged in an overall cross (Figures 1A,B). Thus, the elements of these positive and negative images were “consistent” in their shape. After cows learned these associations, they were presented with “inconsistent” images, where the smaller shapes no longer matched the overall shape (Figures 1C,D). Cows were expected to approach the image if they perceived it to be similar to the original trained positive image. They may approach the image where the overall shape is the same as the trained positive image (“global” element), or they may approach the image where the smaller shapes are the same as the trained positive image (“local” element). For example, consider a cow assigned a circle made of circles as the positive image, and cross made of crosses as the negative image; test images are a circle made of crosses, and a cross made of circles (Figures 1A–D). If this cow approaches the circle made of crosses, this indicates a “global” choice because the cow’s trained positive image (circle) is seen in the overall shape. If this cow approaches the cross made of circles, this indicates a “local” choice because the cow’s trained positive image (circle) is seen in the smaller shapes. Cows that approach the “global” test image more often are interpreted as having a broader attentional scope (“seeing the forest rather than the trees”) while cows that approach the “local” test image more often are interpreted as having a more narrow attentional scope (“seeing the trees rather than the forest”). After completing the attentional scope test, a subset of cows were presented with an image showing only the local element (a single small circle or cross, Figures 1E,F) in the center of the black background. This was to ensure that cows were able to see the small shapes on the screen from the start line. Cows were expected to approach the image matching the trained positive shape, indicating they were able to see single components of the positive image.

**Table 2:** Summary of training and testing steps for dairy cows in the attentional scope task.

Phase (step)	Purpose	Image(s) presented	Criterion to proceed
Initial training			
Habituation	To familiarize cows to the arena and to presentation of food reward underneath the screen	None	Does not move backwards when tray is presented and eats comfortably from tray
Shaping for approach and nose-touch image	To train cows to pay attention to image on screen, and to approach and nose-touch the positive image	Positive	Cow walks to and nose-touches image on screen 10 times without stopping
Discrimination training			
40% negative rate	To train cows to approach and nose-touch the positive image, and to not approach and nose-touch the negative image, at a rate of lower rate of 40% negative and higher rate of 60% positive images	Positive Negative	≥ 80% correct in a single day, up to a maximum of 4 days
50% negative rate	To train cows to approach and nose-touch the positive image, and to not approach and nose-touch the negative image, at equal rates of positive and negative images (50%)	Positive Negative	Average > 80% correct over 2 consecutive days, within 25 d (since start of training)
Attentional scope testing	To test cows’ attentional scope by presenting ‘inconsistent’ global and local images	Positive Global Local Negative	Completed 2 test days. Move to local testing if at least 2 days until forthcoming experiment
Local testing	To verify that cows are able to perceive the small elements of the image from the starting distance	Positive Positive-local Negative-local Negative	

### 2.3. Training and testing apparatus

**Figure 2:**
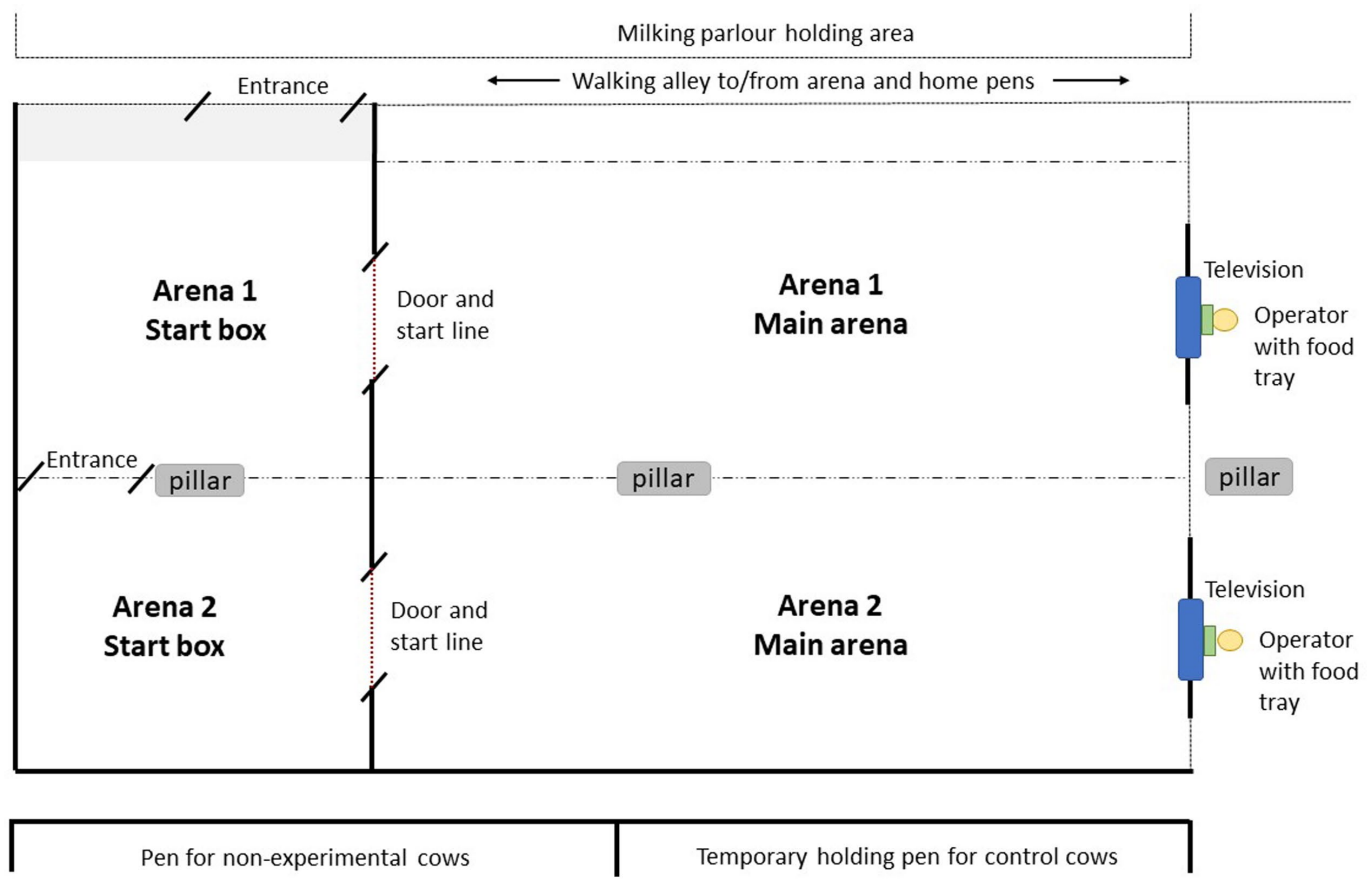
Training and testing facility used for attentional scope procedures (diagram is not to scale). Cows entered along an alleyway into Arena 1 start box, or continued to walk into Arena 2 start box. From the start box, cows crossed the start line into the main arena through a door (*dotted red line*). At the end of the main arena was a display screen (*blue box*), which displayed the positive, negative or test images. An operator (*yellow circle*) sat behind the screen to control the computer which displayed images on the television. The operator also delivered the food reward in a tray (*green box*) by sliding it underneath the television. The sides of the arenas were either solid (*thick black line*), or open-sided moveable fencing (*dotted line*). Arena 1 start box was slightly larger in size due to an entrance area (*shaded gray*). Cows in the start box and main arena were able to see and hear other cows during training and testing.

The training and testing procedures occurred in an arena outlined in Figure 2. For details on dimensions and construction material, refer to Supplementary material. There were two adjacent test arenas (arena 1 and arena 2), each consisting of a start box where the cow was held before entering the main arena. Visual and auditory contact with other cows was still possible from within the arena, but not with their calves. A plywood door allowed access between the start box and the main arena, and cows were able to see over the top of the door while it was closed. At the front of the main arena in front of the start line, a display screen (96.9 × 60.2 × 8.3 cm) was mounted inside a wooden box frame fitted with clear plexiglass for protection. The screen was connected to a laptop computer that displayed the training and testing images with Microsoft Powerpoint (Microsoft^®^ PowerPoint^®^ for Microsoft 365 MSO). An operator sat behind the screen to control presentation of the images from the computer and to deliver rewards and punishments. Cows could see over top and underneath the screen, where the operator’s boots, hands and the inaccessible food tray were visible. A pair of cows from the same treatment pen were held simultaneously in arena 1 and arena 2, with physical contact possible over the fence separating the two start boxes. This was to reduce potential effects of social isolation and allowed for greater efficiency with training. Cows had slightly obstructed views of the adjacent arena while the partner cow was trained, so we do not believe there was social learning of how to respond to images. Images displayed on the screen were similar to visual stimuli reported in Truppa et al. (24), adjusted according to the visual acuity of dairy cows (31, 32). Circle and cross images contained identically sized local (6 × 6 cm) and global elements (42 × 42 cm). Images were viewed by cows from the start line at a distance of 6.2 m (length of main arena).

### 2.4. Initial training

A detailed description of training is provided in Supplementary material.^1^ Each cow was trained by the same experimenter once per day during weekdays for a maximum of 10 min. Before training, cows were feed restricted for approximately 1 h to maintain motivation for the feed reward in the task. Order of training in the arena was maintained within the block.

#### 2.4.1. Habituation

A pair of cows from the same treatment were habituated simultaneously to adjacent arenas, with the screen black. A tray containing two food rewards (familiar TMR on one side, familiar concentrate on the other side) was placed in the center of the arena, which cows could freely approach during 15 min in the arena. On subsequent training days the tray was moved closer to the screen until the cow was comfortably eating with the tray moving in and out from underneath the screen, to familiarize the cow to the delivery of food rewards and to the operator’s hand on the tray. Three cows (two part-time and one full-time) were excluded at this habituation stage.

#### 2.4.2. Shaping

Cows were clicker-trained individually to nose-touch the positive image when displayed on the screen. With the experimenter inside the pen next to the cow in front of the black screen, cows were conditioned to the sound of a click from a clicker device that signaled the immediate delivery of the food tray under the TV (available for 5 s). On subsequent training days, successive approximations of the desired behavior were rewarded with a click and food reward, beginning with small head movements upwards, then head lifted and nose directed to the center of the screen, then finally nose-touch the positive image displayed on the screen (repeated 10 times). Finally, cows had to walk from the start line (6.2 m) to and nose-touch the positive image on the screen, without stopping (repeated 10 times). No cows were excluded at this shaping stage.

### 2.5. Discrimination training

A detailed description of discrimination training is provided in Supplementary material (see Footnote 1). Discrimination training was conducted in pairs (in most cases, the same pair as their habituation day partner) in the same order, with one cow in each arena, for approximately 30 min. A training day always began with 3 ‘refresher’ positive images (to evaluate food motivation), followed by a randomly-selected sequence of 10 images that pseudo-randomly alternated between positive or negative images. Negative image rate was initially 40%, then increased to 50% when cows achieved ≥80% correct responses (out of 10 images), or after 4 training days. This approach was taken due to previous discrimination training experience that animals were more likely to advance if rewards were initially more frequent than punishers. An image was displayed on the screen, and the cow was released from the start box. If the cow did not voluntarily cross the start line, the experimenter pushed her until at least one hoof crossed the start line. Each image was displayed for 30 s after crossing the start line, during which time cows could chose to approach or avoid the image. If the cow correctly approached and touched the positive image, the food reward was delivered for 5 s (Supplementary Video S1; see Footnote 1). If the cow did not approach the positive image, the cow was returned to the start box. However, to facilitate learning during the 40% negative images sequence, the experimenter encouraged her to approach and touch the positive image if she did not go, and the food reward was delivered for 5 s (but this was recorded as incorrect response to positive). If the cow correctly avoided the negative image after 30 s, the experimenter called ‘Good girl!’ and the operator changed the image to black (Supplementary Video S2; see Footnote 1). If the cow incorrectly approached the negative image within 30 s, a punishment was delivered where the operator vigorously waved a small plastic bag attached to a wooden handle 4 times underneath the screen. To account for potential olfactory cues, the food tray was always filled with food and visible to the cow, but it was inaccessible until the cow made a correct response. In between each image presentation in the sequence, the cow was returned to the start box where she waited for approximately 1 min while the cow in the adjacent arena was trained. This “inter-trial interval” is known to increase an animal’s learning speed in discrimination tasks (33); potentially this waiting time could induce frustration or boredom, but the presence of the partner cow was expected to reduce this. There was no wait time in between the 3 “refresher” images, which also served to reduce possible frustration to obtain a food reward at the beginning of the training day. Cows were considered trained and ready for testing when they averaged >80% correct responses (out of 20 images) over 2 consecutive training days. This criterion is intermediate to other attentional scope studies [70% correct for splitfin fish (24); 80% correct for dogs (29); 85% correct for domestic chicks (25)] and is similar to criterion reported for discrimination training in other livestock (34). Three cows required corrective training [following Hintze et al., (35)] which was applied if approach responses to the positive were extinguished for two consecutive training days. Cows were presented a 10-image sequence of all positive images until they approached 5 consecutive images without encouragement (see Supplementary material); two of the three cows met the learning criteria afterwards. Cows needed to complete training (inclusive of initial and discrimination training phases) within 25 days due to enrolment in a concurrent experiment. Three cows (one full-time and two part-time) were excluded because they did not meet the learning criterion by this deadline. A total of 20 cows (8 full-time, 6 part-time and 6 no-contact) completed discrimination training and were eligible for testing.

### 2.6. Attentional scope test

Before beginning the attentional scope test, all 20 cows that completed discrimination training first completed a judgment bias test (JBT) for 4 consecutive days as part of a concurrent experiment; JBT used the same positive and negative training images, but not test images, so it was not expected to interfere with performance in the attentional scope test. This JBT test was intended to verify the effect of experimental housing conditions on emotional state [see Neave et al., (30)]. However, due to the start of a forthcoming experiment, there was limited time to complete attentional scope testing after the JBT. There were 14 cows available to complete attentional scope testing within the required time frame (6 full-time, 6 part-time, 2 no-contact; 9 cows with circle and 5 cows with cross as the positive image). At the time of testing, cows had experienced their housing treatment for about 35 d (mean ± SD; full-time: 33.2 ± 9.0 d, range: 22 to 48 d; part-time: 36.7 ± 4.2 d, range: 31 to 41 d; no-contact: 37.5 ± 10.6 d, range: 30 to 45). Cows were tested in pairs (same partner as during discrimination training) over two consecutive days. To maintain consistency with training, test days always began with 3 consecutive ‘refresher’ positive images (with no inter-trial interval in the start box, identical to training), followed by the test image sequence of 11 images: 4 positive (P), 3 negative (N), 2 global (G) and 2 local (L) images (Figures 1A–D). Thus, over the two test days, cows were presented a total of 8 positive, 6 negative, 4 global and 4 local images. The global and local images were always presented 3rd, 5th, 7th, and 9th in the sequence, and the sequence always ended with a positive image. The test sequence was P-N-G-P-L-N-G-P-L-N-P on day 1, and was N-P-L-N-G-P-L-P-G-N-P on day 2, for all cows. In between each image presentation, the cow was returned to the start box where she waited for approximately 1 min while the cow in the adjacent arena was tested. Positive and negative images continued to be reinforced, but the global and local images were neither rewarded nor punished if the cow approached and touched; in this case, the experimenter called “Okay,” the operator changed the screen to black, and the cow was returned to the start box. This also occurred if the cow did not approach the global or local images after 30 s. A test day lasted approximately 30 min for a pair of cows.

### 2.7. Local test

To determine if cows could distinguish between the local elements of the trained image (serving as a validation of the attentional scope method), cows participated in an additional test after completing the attentional scope test. However, only a subset of 6 cows (3 full-time, 2 part-time, 1 no-contact) completed local testing due to the time constraint that at least 2 days remained until the forthcoming experiment. Cows were tested over two consecutive days with their previous partner during attentional scope testing. Procedures were identical to those during attentional scope testing, except that the test sequence included 2 images of the single local element of the positive trained image (referred to as positive-local) and 2 images of the single local element of the negative trained image (referred to as negative-local) (Figures 1E,F). This testing approach was based on similar procedures by Truppa et al. (24) and Chiandetti et al. (25). Positive-local and negative-local images were neither rewarded nor punished.

### 2.8. Cortisol samples

Saliva samples were collected from each cow before and after each of the two test days to assess possible arousal during the test that may influence attentional scope and thus approach responses to the images. Samples were collected while the cow was restricted with a moveable fence in a corner of the home pen, in the presence of calves. A sample was taken before the cow entered the test arena, between 0730 and 0800 h (cows had been with their calves for at least 2 h since returning from morning milking). A second sample was taken 20 min after the cow left the test arena, which corresponds to the time lag of peak cortisol concentration in saliva after a stressful event in dairy cows (36). Saliva was collected from the cow using a synthetic swab (Salivette^®^ Cortisol, Sarstedt AG & Co., Numbrecht, Germany) held with metal forceps and placed in the inner cheek for at least 30 s. The swab was then placed in the collection tube (Salivette^®^ Cortisol, Sarstedt AG & Co., Numbrecht, Germany) and immediately placed on ice. Swabs were centrifuged for 20 min at 4°C at 1500 x g. Supernatant was transferred to 1 mL cryotubes in four 55 μL aliquots and frozen at −20°C until laboratory analysis. Saliva samples were thawed and analyzed for free cortisol concentrations by ELISA (Saliva Cortisol Enzyme Immunoassay Kit No. 1–3,002, Salimetrics, Carlsbad, CA, United States), running each sample in duplicate. Samples from the same individual were analyzed on the same assay plate to minimize the influence of inter-assay variation according to the within-subject sampling test design, and the coefficient of variation between duplicates was ≤9.2%. Two cows had cortisol samples taken on only the first test day (1 full-time and 1 part-time), and one cow could not be sampled on either test day (1 part-time) due to safety issues at sampling.

### 2.9. Data recording and statistical analysis

The cow’s response to each image was recorded by the experimenter as Go (approached and touched within 30 s, coded as 1) or No-Go (did not approach and touch within 30 s, coded as 0) in a notebook immediately after each image presentation during training and testing days. The 3 consecutive “refresh” positive images that preceded a training and testing sequence were not included for analysis. Uncertainty about response records were verified from the video camera (Hikvision DS-2DE2A204IW-DE3) mounted above each arena. Due to the go/no-go design of the attentional scope task, cows could approach only global, only local, both global and local, or neither of the images. Therefore, cows were categorized based on their approach responses to the four presentations of global and local images. The number of cows from each housing treatment in each category (only global, only local, both or neither) were tallied. Due to low sample size for no-contact cows that completed attentional scope testing (*n* = 2), statistical comparisons of treatment were restricted to full-time and part-time (*n* = 6 each); results for no-contact cows are presented descriptively. All statistical analyzes were performed using SAS Studio (OnDemand for Academics, SAS Institute Inc.). All outcome variables were assessed for approximation of a normal distribution using PROC UNIVARIATE and examining model residuals; no transformations were required. For all models described below, the degrees of freedom method was based on Satterthwaite approximation and backwards elimination of explanatory variables was performed until all remaining variables in the model were *p* < 0.3. Statistical significance was declared at *p* ≤ 0.05.

We tested whether the number of days to complete discrimination training was affected by the fixed effects of housing treatment and assigned positive symbol, including parity (primiparous or multiparous) as a fixed effect and cow ID within block as a random effect (general linear mixed effects model; PROC MIXED). Parity was removed from the final model after backwards elimination. As an indicator of arousal, we tested whether cortisol concentration was affected by the fixed effects of treatment, phase (before or after test), and test day (1 or 2); parity (primiparous or multiparous) was included as a fixed effect, and cow ID within block was a random effect, and included repeated observations of cow on test days (general linear mixed effects model; PROC MIXED). The only fixed effects retained in the model after backwards elimination were treatment, phase and parity. Because cortisol concentration did not change from before to after the test, but depended on treatment (see Results), the cortisol concentration before the test was used as a covariate in further models to control for arousal related to housing condition.

To address the main study objective, a binary logistic regression model with logit link and binomial distribution (general linear mixed effects model; PROC GLIMMIX) tested whether the logit of Go-responses in the test phase differed between global and local images, and whether this depended on housing treatment. The outcome variable was Go-response (1 = approached; 0 = did not approach), and fixed effects included image (positive, negative, global or local), treatment (full-time or part-time), parity (primiparous or multiparous), test day (1 or 2; to evaluate learning over repeated days), arena (1 or 2; because arenas were not identical), cortisol concentration before testing (to account for possible arousal influencing approaches to images), and days to complete discrimination training. The interaction of treatment × image was not possible to test because part-time cows never approached a global or negative image (see Results). To account for the repeated observations of image within each test day per cow, these were set as random effects with cow ID within block as the subject. Test day and parity were removed from the final model after backwards elimination. In a further exploratory analysis, cows were categorized as having only ever approached the global image, only ever approached the local image, approached both, or approached neither. The difference between housing treatments in the number of cows in each category was tested using a Fisher exact test (PROC FREQ).

For the local test phase (*n* = 5), the Go-response rate (percentage of images approached out of 4) was calculated for the positive-local and negative-local images across the two test days for each cow. A mixed regression model (PROC MIXED) tested whether cows more often approached the positive-local image than the negative-local image in the local test phase (indicating they could distinguish between the local elements of the trained image). The outcome variable was the Go-response rate; fixed effects were limited to image (positive-local or negative-local), and treatment (full-time or part-time) due to the low sample size; image was the only variable retained in the model after backwards elimination. Cow ID within block was included as a random effect.

## 3. Results

Statistical comparisons of full-and part-time cow-calf contact treatments (*n* = 6 each) are presented, but only descriptive results for no-contact treatment due to low sample size (*n* = 2). The cows that completed attentional scope testing (*n* = 14) required (mean ± SD) 3.0 ± 1.1 d (range 2–5 d) to complete the habituation phase, 7.0 ± 2.2 d (range 4–11 d) to complete the shaping phase, and 8.1 ± 3.5 d (range: 4–15 d) to complete discrimination training. There was no difference in the number of days to complete discrimination training between the two different positive trained images (circle: 7.6 ± 1.4 d; cross: 9.9 ± 1.6 d; F_1,9_ = 1.3; *p* = 0.29), or between housing treatments (full-time: 8.7 ± 1.5 d; part-time: 8.8 ± 1.5 d; F_1,9_ = 0.0; *p* = 0.96). Cortisol concentration was higher in full-time compared to part-time cows (0.19 ± 0.01 μg/dL versus 0.14 ± 0.02 μg/dL, respectively; F_1,8_ = 6.9; *p* = 0.03). Cortisol did not differ before versus after the test (F_1,10_ = 1.2; *p* = 0.30) or between test days (F_1,8_ = 0.6; *p* = 0.46), indicating that testing itself did not affect arousal.

In the local test, cows were able to perceive the local elements of the trained positive and negative images and respond correctly when only a single local image was presented; cows in the full-and part-time treatments (*n* = 3 and 2, respectively) correctly approached the positive-local more often than the negative-local image (85.0 vs. 25.0 ± 13.2% of images approached, respectively; F_1,4_ = 10.3; *p* = 0.03). The one no-contact cow went to 100% of the positive-local and 25% of the negative-local images. Thus, this test served as a validation of the images used for the attentional scope test.

**Figure 3:**
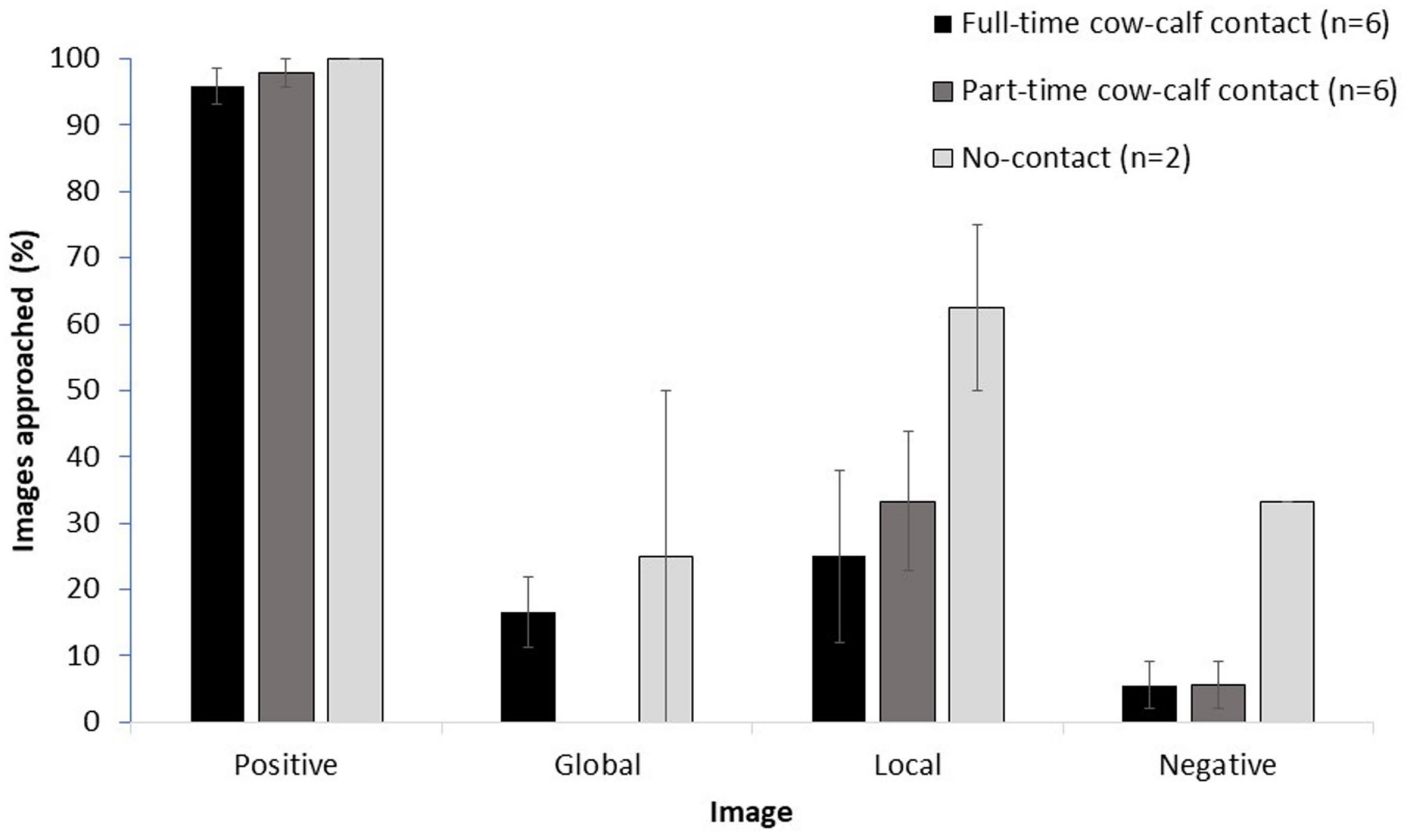
Raw mean (± SE) percentage of images approached during the test phase for all tested cows in each cow-calf contact treatment (6 full-time, 6 part-time, 2 no-contact). Cows were trained to approach the positive image and to avoid approaching the negative image. Approaches to global and local images were not reinforced. Each day, for 2 days, cows were presented a pseudo-random sequence of 10 images: 4 positive, 2 global, 2 local and 3 negative. Cows had 30 s to decide to approach the image.

In the attentional scope test, housing treatment affected the likelihood of approaching an image, where full-time cows approached images more often than part-time cows (odds ratio (95% CL): 4.4 (1.1–17.5); t_1,20.2_ = 2.2; *p* = 0.04). Cows from all treatments (*n* = 14) continued to approach the positive images and avoid the negative images (mean ± SE: 97.3 ± 1.4% versus 9.5 ± 3.4% of images approached, respectively), and approached fewer global (10.7 ± 4.3%) and local images (33.9 ± 7.7%) than the positive image (Figure 3). Statistical comparison of full-and part-time cows only (*n* = 6 each) showed that they were more likely to approach the local than the global image (odds ratio (95% CL): 4.7 (1.3–17.1); *t*_1,245_ = 2.4; *p* = 0.01). Cortisol concentration before testing did not affect approach responses to images (F_1,24.9_ = 3.9; *p* = 0.06).

**Figure 4:**
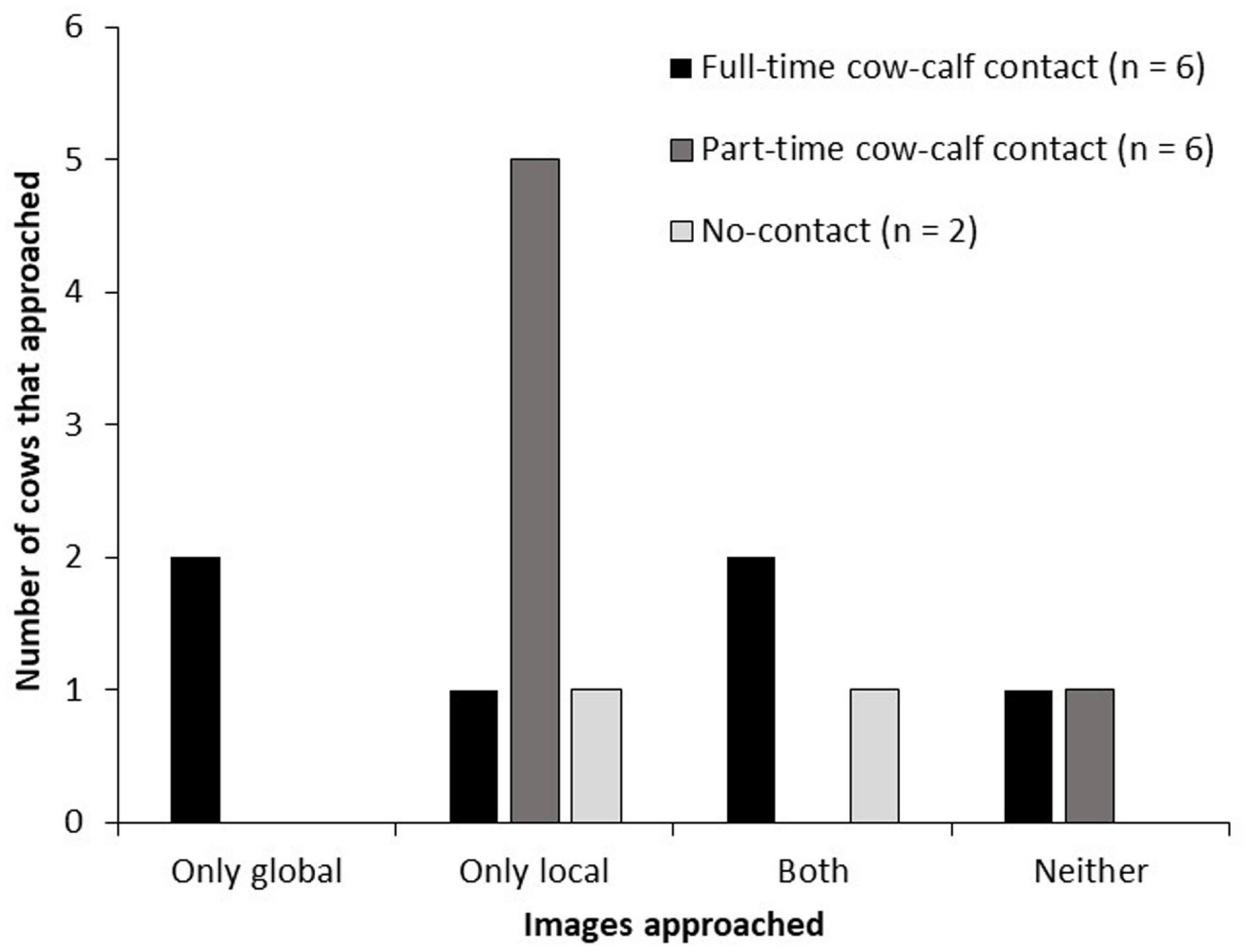
Number of tested cows in each cow-calf contact treatment (6 full-time, 6 part-time, 2 no-contact) that approached only the global image, only the local image, both global and local images, or neither global nor local image. Cows were trained to approach the positive image and to avoid approaching the negative image. Approaches to the global and local images were not reinforced. Each day, for 2 days, cows were presented a pseudo-random sequence of 10 images: 4 positive, 2 global, 2 local and 3 negative. Cows had 30 s to decide to approach the image.

When cows were categorized according to their responses to the images, there was an effect of housing treatment on the number of cows that approached the global and/or local images (Figure 4). Part-time cows never approached a global image, and more part-time than full-time cows exclusively approached the local image (5 versus 1 of 6 cows, respectively; *p* = 0.01). An equal number of part-time and full-time cows (1 of 6 cows) approached neither of the global or local images. One no-contact cow approached only local images, and the other no-contact cows approached both global and local images.

## 4. Discussion

This proof-of-concept study explored whether cows show differences in attentional scope (i.e., attentional processing of visual hierarchical images) depending on housing conditions that were verified for inducing differences in emotional valence (i.e., relative positive or negative states). We observed that cows more often approached the local than the global image (i.e., “see the trees before the forest”), especially in cows managed part-time with their calves; however approach responses to global and local images were low, which may be related to methodological constraints or learning ability. Statistical comparison with the no-calf-contact treatment was not possible due to low sample size. These results are interpreted in light of the human literature showing differential allocation of attention depending on emotional state. Refinements to methodology and study design are also discussed to further the development of attentional scope in the study of animal emotions.

### 4.1. Attentional scope

Dairy cows in our study were more likely to approach the local image, although it must be noted that approach responses to both global and local images were low. Other studies have also found that animals tend to show a local bias in similar tasks, including in domestic chicks (25), pigeons (26), dogs (29) and several non-human primates (37, 38). A local bias may occur if animals have difficulty with processing the overall global arrangement of shapes. For instance, this probably requires first identifying the smaller local elements and visually connecting them to form the overall global shape matching the local element (26, 39). We verified that cows were able to correctly distinguish the single local elements of the positive and negative images, but we did not present a test for the global elements [see Chiandetti et al. (25) for an example, although this introduces solid lines that have never been viewed before]. A test for distinguishing global elements would help to establish if this bias was related to learning or processing difficulties with these complex hierarchical images. Although the size and spacing of the shapes in our study were based on previous studies of visual acuity of cattle in discrimination tasks (31, 32, 40), the attentional scope test herein presented more complex images than those studies. Furthermore, the size and density of the shapes in the images may affect attentional bias (22, 39, 41), although this was not observed in other studies (24, 26, 27, 42).

Aside from possible learning or processing difficulties and the size or density of the images, the observed local bias could be related to differences in attentional processing, arising from directed attention toward the narrowed rather than broadened visual field. If indeed this is occurring in cattle, this may relate to their adaptive vision featuring a narrow binocular field (30 to 50°) (43) and a retina with a horizontal ‘visual streak’ with heightened visual acuity that is thought to be adaptive for detection of predators on the horizon (28), and allow optimal visual discrimination of objects from a distance (44). Our dairy cows viewed images from a distance positioned at eye-level in the center of the visual field, which may have promoted processing of the details of the image. For example, in humans, stimuli presented at the center of the visual field led to a local bias (45). Although the two types of stimuli in our study (cross and circle) did not affect how cows responded, future studies using other stimuli may need to consider these possible influences on visual processing. As a grazing species, the evolutionary history of attending to global and local visual stimuli remains unclear. Overall, the low response rate to both global and local images (11 and 34%, respectively) requires caution in drawing conclusions about a true attentional scope bias in cows, since methodological or learning issues cannot be ruled out.

The influence of emotional state on attentional scope has been debated extensively in the human literature, with divergent conclusions including the role of arousal or motivational intensity in driving biases (reviewed by Vanlessen et al. (46) and Lacey et al. (21)). Only recently has this relationship been explored in animals (29). Those authors reported mixed results, where dogs receiving food rewards showed a narrowed attentional scope when in an induced positive state, regardless of arousal intensity, but the opposite was observed when dogs received social rewards. The latter result, where a narrowed attentional scope occurred during a negative emotional state, aligns with a number of studies in humans (16, 47–49). Therefore, we hypothesized that cows with only part-time calf contact, or with no calf contact, may show a narrowing of attentional scope (i.e., local bias) suggestive of a negative, or less positive, emotional state, relative to cows with full-time calf contact; this prediction is grounded in findings of our previous study indicating differences in emotional valence between full-and part-time calf contact using a validated judgment bias test (30). Our results revealed that part-time cows exclusively approached the local image (suggestive of a local bias), while full-time cows approached both global and local images. This finding in the full-time cows possibly suggests a level of attentional flexibility, which authors have noted may arise from positive emotional states that promote wider processing of information from the environment (17, 48). Alternatively, the global and local images may have been perceived as ambiguous, since the cows had never seen this combination of shapes before; thus, full-time cows may have tried a more ‘optimistic’ strategy by occasionally approaching the global image in an attempt to obtain a reward, similar to how animals respond in a judgment bias task (4, 10). A series of previous studies in humans has shown that positive mood enhances the ability to see alternative cognitive perspectives and generate innovative solutions to problems [reviewed in Ashby et al. (50)]. On the other hand, negative mood is suggested to induce inhibitory control of attention, presumably as an adaptive process to restrict information input to only relevant, possibly threatening, stimuli (48, 51). The low response rate to local and global images in both treatments requires cautionary interpretation of biases related to emotional state arising from our housing conditions. Furthermore, we were unable to statistically compare the no-contact treatment.

Arousal or high motivational states may affect how attention is allocated [see review by Lacey et al., (21)]. For example, participants experiencing a high versus low intensity positive mood state (desire versus amusement) were more likely to choose the local over the global image (52). However, the attentional scope study in dogs observed no difference in local image choices between the high and low arousal positive affect conditions which were verified using heart rate as a measure of arousal (29). We attempted to control for possible arousal affecting image choices in the test by measuring salivary cortisol concentration in the cows before and after the test; indeed, we found no relationship between cortisol concentration and whether cows approached the images, suggesting attentional scope was not affected by cortisol (and thus arousal). Similarly, exogenously administered ACTH to sheep (leading to increased plasma cortisol concentration) did not impact attention or judgment biases (7). In addition, cortisol concentration did not change after completing the test, suggesting that temporary separation of cows from their calves to participate in the test was not especially arousing. However, full-time cows had higher baseline cortisol concentrations than part-time cows, which was unexpected considering that part-time cows experience longer periods of separation from their calf that could increase baseline cortisol concentrations. A possible explanation is that full-time cows had greater opportunities to nurse during the day and nighttime, which is known to increase cortisol in suckling cows (53). Nonetheless, we found no relationship between cortisol concentration and whether cows approached the images. Therefore, both our study using salivary cortisol concentration, and Hamlaoui et al. (29) using heart rate, do not point to biases in attentional scope being linked merely to arousal level.

### 4.2. Cow-calf contact

Our housing conditions differed in the duration of cow-calf contact, which may have contributed to biases in attentional scope due to differences in emotional state. There are a couple lines of very recent evidence that support our expectation that cows with full-versus part-time calf contact may differ in emotional state. Our recent study on the same cows used a validated judgment bias methodology to show that part-time cows had a negative bias relative to full-time cows (but not different from no-contact cows), suggesting a more negative (or less positive) emotional state (30). Behavioral evidence also suggests that cows managed part-time with their calves on pasture are more restless at milking (i.e., more stepping and kicking), which was interpreted as a negative behavioral response to periods of separation from their calves (54). However, conditions other than duration with the calf may have contributed to emotional state differences in our study, such as different lying surface during the nighttime; part-time cows moved to a free-stall pen with mattresses while full-time cows remained in the straw-bedded pen (a separate identical deep-bedded area for part-time cows at night was not possible due to research facility constraints). Dairy cows show strong preferences for deep-bedded over free-stall areas (55), and will work to access a straw-bedded over a mattress lying surface (56). Overall, the series of disturbances experienced by the part-time contact treatment, including repeated separations from their calves overnight, change of pen and change of lying surface, likely contributed to these lines of evidence suggestive of a negative emotional state.

In general, cow-calf contact itself is expected to lead to positive emotional states, but work to date provides mixed support from behavioral indicators (such as play, exploratory and grooming behaviors; see Keeling et al. (57) for a review of positive welfare indicators in cattle). For instance, Waiblinger et al. (58) observed greater play in calves when the cow was present, but not Bailly-Caumette et al. (59), and exploratory and self-grooming behavior did not differ between calves with or without access to cows (60, 61). The focus of these studies was on the calf, with no previous study examining indicators of positive states of cows in these systems. It is known that cows are motivated to gain access to their calves (62, 63), suggesting that cow-calf contact is likely to be experienced as positive; however, meeting a behavioral need does not by default promote positive emotional states, which were not measured in these studies. We encourage future studies to better explore the expression of positive emotional states in cow-calf contact systems (compared to a no-calf-contact condition) by employing a combination of cognitive, behavioral and physiological indicators, which could be used to determine the validity and reliability of a refined version of the attentional scope test developed in this study.

### 4.3. Methodology limitations

An unexpected major limitation is that cows had low approach response rates to the global and local images, making specific conclusions regarding attentional scope difficult. The go/no-go task design permitted a no-go response, allowing the cow to avoid both global and local images (i.e., only one image was presented at a time and the cow could decide to approach it or not). In contrast, previous attentional scope studies in animals used an active choice (go/go) design, where the contrasting images were presented simultaneously and the animal had to make an active choice between the two [e.g., (23–25, 29)]. We used a go/no-go task because it was used in previous studies in dairy cattle involving discrimination training (64), including visual tasks (65, 66); active choice discrimination method can take longer to train (34, 35), possibly because it is more cognitively demanding to learn (2). However, a disadvantage of our task is that the same response to both global and local images is difficult to interpret; this issue has also been raised with regards to both go/no-go (67) and go/go judgment bias methodologies (68).

One possible explanation for the low approach responses to both global and local images is that cows did not perceive either to resemble the positive training image. The use of a discrimination task to assess attentional scope means that global and local choices are a result of previous learning (23), so cows may have learned (attended to) both local and global elements of the positive training image rather than one or the other; thus, when these elements were put in contrast during testing, the cows were less likely to approach because this image did not exactly match the positive image they had learned. This proposition would suggest that perceptual learning of global versus local elements of two-dimensional stimuli is not so straightforward; for instance, pigeons were shown to rapidly shift attention between global and local elements in a similar task to ours, demonstrating that some animals may possess dynamic attentional processing that evolved to suit complex natural environments (69).

A second explanation is that cows may have attended to the negative elements contained in the global and local images, and thus avoided approaching to prevent receiving the expected punishment. This methodological complication limits our ability to determine toward which aspect (global or local) of the training image (positive or negative) that the cow showed a bias. A solution would be to substitute completely novel shapes to form the global versus local element [e.g., see (29)], although it is unknown if this novelty induces some aversive response. The salience of the punisher (waving plastic bag) could also have contributed to greater avoidance of global and local images; less salient punishers in discrimination tasks, like withholding the reward (10) or delaying the next image presentation (65) may improve response rates to the global and local images.

Third, cows could have learned not to approach after repeated presentations of global and local images since they were not reinforced; this is a common issue described in judgment bias studies in farm animals [e.g., dairy cattle: (64); sheep: (70); pigs: (71)]. Although, our analysis did not reveal an effect of test day, with responses to test images remaining below 30% on both test days, the JBT task experienced prior to the attentional scope task also did not reward test images; this may have contributed to reducing response rate to the test images.

### 4.4. Other limitations

An obvious limitation of our study is the low sample size of 6 cows per full-and part-time contact treatment (and 2 cows of the no-contact treatment that were presented descriptively); this resulted in large variability in responses to the global and local images. This low sample size was not due to failure to learn the discrimination task; 20 of 30 cows that were enrolled for training successfully learned the discrimination task in the given time frame (by comparison, 10 of 21 dogs learned the task in Hamlaoui et al., (29)). A time constraint of a forthcoming experiment reduced our sample size to 14; possibly if no-contact treatment cows had learned the discrimination task quicker we could have achieved a greater sample size. However, our population sample was still biased to those individuals that were able to learn in the available time frame. Furthermore, due to differences in learning time, some cows were tested earlier than others and therefore had also experienced their treatment for less time. This could contribute to differences in responses at test, but differences in training time (and thus time experiencing the treatment) were accounted for in our model. A stronger experimental approach to minimize variability would be to use a within-subjects design where each cow experiences both treatments; however, cross-over designs with cow-calf contact housing are impractical and experience with the previous treatment is likely to heavily impact response to the subsequent treatment. Therefore, our conclusions regarding attentional scope and emotional state in cows housed full-or part-time with their calves remain very cautious in light of the methodological and sample size limitations. Future use of the attentional scope methodology should first consider refinements in the areas described above, and include complementary behavioral or physiological measures of emotional valence and arousal (such as cortisol or heart rate) to confirm if attentional scope biases are indeed driven by affective valence and/or arousal as they appear to be in humans.

Finally, previous attentional scope studies in animals, and indeed most other cognitive methods in livestock, tested animals individually and usually separate from their home environment. The social isolation of herd animals in these tests could result in a negative affective state unrelated to the cow-calf contact treatment, which the current study attempted to mediate by training and testing cows in pairs (visual, auditory and physical contact from the start box of the test arena). A drawback of testing in the presence of a social companion is that some uncontrollable distractions are introduced (e.g., sounds from the partner cow) that may have affected how cows responded to the global and local images. However, we argue the advantage of testing a herd species with a familiar social partner outside of the home environment (limiting negative emotional state due to social isolation) likely outweighs the disadvantage of possible distraction. Despite our attempts to limit social isolation, cows were still temporarily separated from their calves at testing, which may have affected the responses to images (although arousal, measured by cortisol, did not affect these responses). We encourage future studies of emotional state in cattle and other livestock to consider the social environment during testing.

## 5. Conclusion

This proof-of-concept exploratory study investigated attentional scope in livestock using a novel methodology. Dairy cows managed part-time with their calves showed a bias toward the local elements of an inconsistent image (a narrowing of attentional scope), while cows with full-time contact with their calves were more mixed in their approaches to the global and local elements of inconsistent images. Based on evidence from human literature, this narrowing of attentional scope would suggest a more negative (or less positive) emotional state in part-time cows relative to full-time cows, but we were unable to statistically include the no-contact treatment for comparison. However, approaches to the inconsistent images were low which makes conclusions regarding attentional scope difficult. Refinements to the attentional scope methodology and sample size are necessary to evaluate the validity and reliability of attentional scope in assessing emotional states of cows in different management conditions.

## Data availability statement

The data presented in this study are deposited in the Mendeley Data repository, accession number: https://data.mendeley.com/datasets/rhdc3hmwcy/1.

## Ethics statement

The animal study was approved by Danish Animal Experiments Inspectorate (Permit No. 2021-15-0201-00989). The study was conducted in accordance with the local legislation and institutional requirements.

## Author contributions

HN: Conceptualization, Data curation, Formal analysis, Investigation, Methodology, Writing – original draft. J-LR: Funding acquisition, Methodology, Project administration, Resources, Supervision, Writing – review & editing. MB: Funding acquisition, Methodology, Resources, Supervision, Writing – review & editing. EJ: Investigation, Writing – review & editing. MJ: Funding acquisition, Methodology, Project administration, Resources, Software, Supervision, Writing – review & editing.

## Footnotes

^1^ https://data.mendeley.com/datasets/rhdc3hmwcy/1
