# Supplementary material for: Do cows see the forest or the trees? A preliminary investigation of attentional scope as a potential indicator of emotional state in dairy cows housed with their calves
Source: Front Vet Sci. 2023 Sep 28;10:1257055. doi: 10.3389/fvets.2023.1257055 (PMC10568025; doi:10.3389/fvets.2023.1257055)
Supplement: Supplementary file 1 [file Data_Sheet_1.docx]

Supplementary Material:

Detailed description of training methodology

# Initial training phase

Briefly, each cow was trained once per weekday for a maximum of 10 min, and experienced approximately 1 hour of feed restriction beforehand (feed trough blocked with plywood) to maintain motivation for the feed reward in the task. Cows were moved from the home pen to the arena in pairs for habituation, and individually for subsequent training days. Order was maintained throughout training, and by treatment (e.g. part-time cows first, full-time cows second), which rotated between blocks. Each training day began at the step that the cow had previously achieved, and the experimenter attempted to advance the cow thereafter. The experimenter stood inside the arena with the cow during training, and the same experimenter trained all cows. Initial training days were terminated when the cow met the step’s learning criteria, or after a maximum of 10 min.

## Step 1. Habituation

A pair of cows were habituated simultaneously to adjacent arenas. Cows were alternately assigned to arena 1 or arena 2 within treatment. A divided black baking tray (36.5 × 49.0 cm) containing two food rewards (familiar TMR on one side, familiar concentrate on the other side) was placed in the center of the arena. The cow could freely approach and eat the food during 15 min in the arena. The television screen remained black. Vocalizations were recorded by two observers standing visible behind the television screens. Cows that did not eat from the tray and had a high rate of vocalizations repeated this habituation day. On subsequent training days (each day to a maximum of 10 min of individual training in the arena), the food tray was moved closer and closer to the television screen until the cow was comfortably eating underneath it. The operator behind the television then began to move the tray in and out from underneath the television, such that the tray became briefly inaccessible; this familiarized the cow to movement of the tray and the presence of the operator’s hand on the tray. A second experimenter stood inside the arena with the cow to observe behavior and instruct the operator. Cows advanced to the next step when they did not react negatively to the movement of the food tray (often by stepping backwards or turning away) and ate comfortably when the food tray was accessible. There were 3 cows that were excluded at this stage (2 part-time who never ate from the tray after 5 or 6 days; 1 full-time who progressed to eating from the tray, but would not eat under the TV after 10 days).

## Step 2. Shaping to nose-touch image

Cows were clicker-trained to nose-touch the positive image when displayed on the television (Supplementary Video 1). The experimenter stood inside the pen with the cow standing in front of the television with black screen. Cows were conditioned to the sound of a click from a clicker device (AniOne Basic Clicker, MultiFit Tiernahrungs GmbH, Krefeld, Germany) by initially pairing a click with the immediate presentation of the food reward under the television (repeated 10 times). Cows could consume either or both food rewards for 5 seconds, after which the food became inaccessible and was replenished by the operator.

Next, the cow needed to lift her head up to the centre of the television screen before receiving the click and food reward. This was achieved by initially rewarding small head movements upward, then rewarding when eyes were level with the bottom of the screen, and then centered to the television screen. Next, the assigned positive image was displayed on the screen and the cow needed to nose-touch the centre of the screen to be rewarded; this was repeated 10 times. Assignment to circle or cross positive image (Fig. 2 a-b) was alternated between treatments and arenas.
 Finally, cows were trained to walk toward and nose-touch the positive image on the television. This was done by initially requiring the cow to walk a short distance to the television screen (approximately 4 m; repeated 5 times), then requiring the cow to walk the full distance (6.2 m from the start line in the start box) to the television screen. Cows received the click and food reward if they approached and nose-touched the image. Cows had to walk, without stopping, from the start line to nose-touch the positive image 10 times before advancing to the discrimination training phase. No cows were excluded at this stage of training.

# Discrimination training phase

Discrimination training days were conducted in pairs (in most cases, the same pair as their habituation day partner), with one cow in each arena, for approximately 30 min. This was to reduce potential effects of social isolation and allowed for greater efficiency with training. Order of training was the same as for initial training phase, and cows experienced approximately 1 hour of feed restriction prior to training. A training day always began with 3 ‘refresher’ positive images, followed by a randomly selected sequence of 10 pseudo-randomly alternating positive or negative images (always beginning and ending with a positive, and no more than 2 consecutive positive or negative images; example sequences: P-N-N-P-P-N-N-P-N-P or P-N-P-P-N-N-P-N-N-P). Twelve possible sequences met these criteria, of which one was randomly selected each training day and used for all cows on that day. Initially the sequence was 40% negative – 60% positive images, then increased to 50% negative and positive images.
 To begin a training sequence, the door into the main arena was opened, the image was displayed on the television, and the cow was released from the start box. If the cow did not voluntarily cross the start line, the experimenter pushed her until at least one hoof crossed the start line. Each image was displayed for 30 s, and cows chose to approach or avoid the image. The consequences of the cow’s response to the positive and negative images are outlined in Figure S1. When a cow approached and touched the positive image (correct response to positive), the food reward was delivered for 5 s (food reward was replenished after each presentation) (Supplementary Material Video 1). When a cow did not approach the positive image (incorrect response to positive), the experimenter encouraged her to approach and touch, then the food reward was delivered for 5 s. When a cow did not touch the negative image (correct response to negative) within 30 s, the experimenter called ‘Good girl!’ and the operator changed the image to black (Supplementary Material Video 2). When a cow approached and touched the negative image (incorrect response to negative), the punishment was delivered (the operator vigorously waved a small plastic bag attached to a wooden handle 4 times underneath the television). To account for potential olfactory cues, the food tray was always filled with food and visible to the cow, but it was inaccessible until the cow made a correct response. In between each image presentation in the sequence, the cow was returned to the start box where she waited for approximately 1 min while the cow in the adjacent arena was trained. This ‘inter-trial interval’ is known to increase an animal’s learning speed in discrimination tasks (Ward et al., 2013).

*
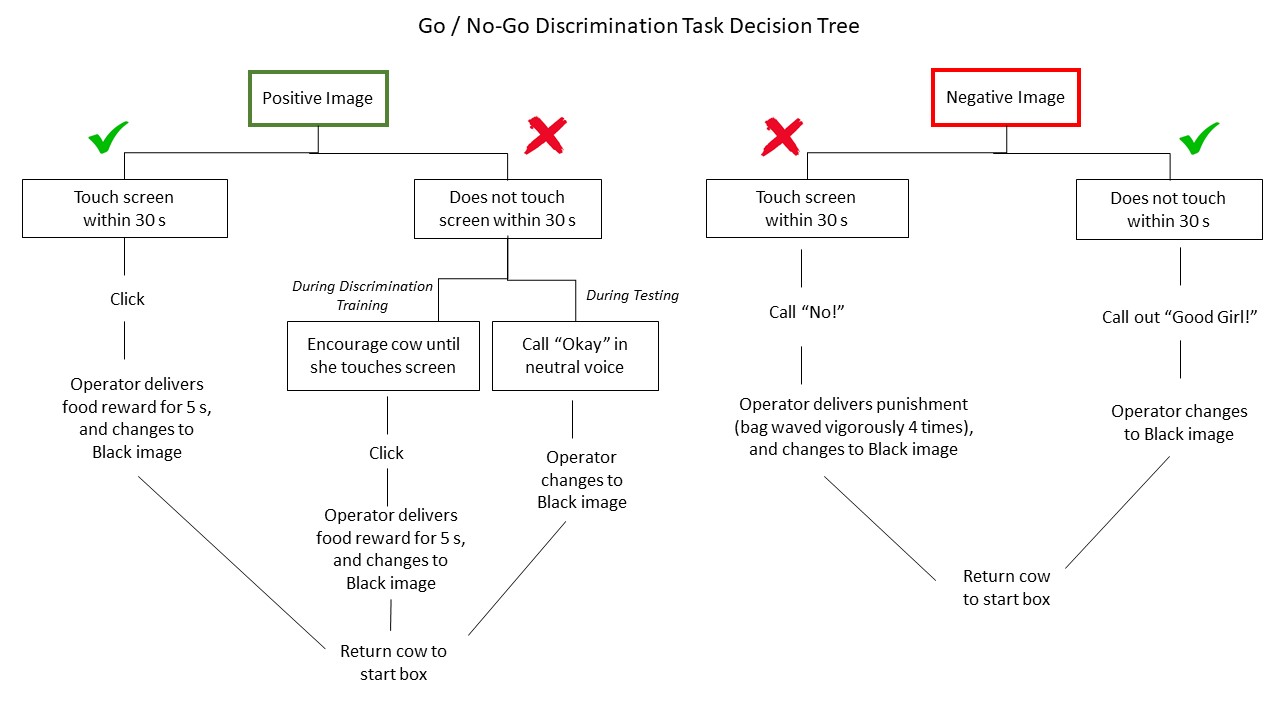
****Figure S1.*** *Decision tree outlining the consequences of a cow’s response to each of the positive and negative images during discrimination training and testing.*

Some cows responded more negatively to the punishment than others, resulting in extinguished approach responses to the positive image. If this occurred for two consecutive training days (i.e. cow never approached the positive image on her own), then a ‘positive correction’ day was introduced, which is commonly used to correct for biases toward specific stimuli (following Hintze et al., 2017). Instead of the normal training sequence of positive and negative images, the sequence was 10 consecutive positive images. If cows began to approach and stopped, they were encouraged to continue approaching and nose-touch. Cows advanced to the normal training sequence of positive and negative images after completing 5 consecutive positive images on their own (allowing 30 s per image). There were three cows that required ‘positive correction’ (one full-time, and one part-time cow for seven days, and one part-time cow for one day), of which the latter part-time cow was excluded due to lack of time to complete training.
 When cows achieved ≥ 80% correct responses (out of 10 images) with 40% negative images in the training sequence, or after four training days, they advanced to 50% negative images in the training sequence. Cows were considered trained and ready for testing when they averaged > 80% correct responses (out of 20 images) over 2 consecutive training days. Cows needed to complete training (inclusive of initial and discrimination training phases) within 25 days due to enrolment in a concurrent experiment. Three cows (one full-time, and two part-time cows) were excluded because they did not meet the learning criterion by this deadline.
